# A family practice breastfeeding education pilot program: an observational, descriptive study

**DOI:** 10.1186/1746-4358-2-4

**Published:** 2007-03-05

**Authors:** Christine M Betzold, Kathleen M Laughlin, Carol Shi

**Affiliations:** 1 Children's Hospital of Orange County, Orange, California, USA; 2 Newport Family Medicine, Newport Beach, California, USA

## Abstract

**Background:**

The United States Preventive Services Task Force found that effective interventions for extending breastfeeding duration are generally begun during the prenatal period, provide ongoing support for patients and combine information with face-to-face guidance. A 2001 literature review had similar findings but also found that employing a lactation consultant in the clinical setting may increase breastfeeding duration rates. Thus, a program was developed at a family practice office that employed a lactation consultant and followed the American Academy of Pediatrics' "Ten Steps to Support Parents' Choice to Breastfeed Their Baby."

**Methods:**

The program distributed handouts at each prenatal and well-child visit (up to one year). Using questionnaires, a small audit project evaluated the program's impact on breastfeeding goals, duration, in-hospital exclusivity and maternal perception of success. Mothers completed goal surveys at baseline and post-intervention, usually while waiting for prenatal clinic visits. Duration was assessed by surveys completed during well-infant visits, postal mailings or telephone interviews at breastfeeding cessation, 6 months and 1 year. The outcomes measured were increases in goals, maternal perception of success, duration and in-hospital exclusivity.

**Results:**

Participants included 33 women: 48% had a bachelor's or master's degree, 61% were non-Hispanic white, and 67% reported incomes of US$75,000 or higher. At baseline 5/31 planned to exclusively breastfeed for 4–6 months and 5/33 planned to breastfeed for 6–12 months. Post-intervention there was a 200% increase (15/31) in the exclusively breastfeeding 4–6 month group and a 160% increase (13/33) in the 6–12 month duration group. Actual in-hospital exclusivity rates were 61%, 6-month duration rates were 73%, and 12-month rates were 33%. Over 75% of mothers reported feeling successful.

**Conclusion:**

This small pilot educational program may have significant impacts on breastfeeding goals. Setting and meeting goals may increase duration and in-hospital exclusivity rates as well as enhance maternal self-perception and empowerment due to succeeding at their breastfeeding goals and/or experiencing a fulfilling breastfeeding relationship.

## Background

The World Health Organization recognizes the importance of promoting and supporting breastfeeding as the optimal feeding method used exclusively for at least 6 months and continued along with complementary feeding for no less than two years of life [1]. Given that current overall United States (U.S.) breastfeeding rates fall short of these recommendations [2-5], implementation of programs that promote breastfeeding are indicated. Additionally, for breastfeeding programs to be optimally effective, they should be combined with:

1. "Baby-Friendly" hospital practices [1,6]

2. Primary care settings which follow the AAP Task Force on Breastfeeding's "Ten Steps to Support Parents' Choice to Breastfeed Their Baby" (AAP's 10 Steps) [7]

3. A variety of other outpatient interventions [8,9].

While there are a number of ways to implement breastfeeding promotion within a family practice, this family practice in the U.S. developed a small pilot program consisting of a variety of interventions. The practice's program aimed to increase breastfeeding goals and document the participant's breastfeeding rates. There is compelling evidence that breastfeeding goals affect duration; however, research is needed to identify whether education can increase maternal goals [10]. The authors aimed for participants to meet or exceed local breastfeeding rates and the Healthy People 2010 goals [3,4]. This paper reports on the program.

Breastfeeding rates collected by the state of California varied within the county of Orange. In-hospital 2002 initiation rates ranged from 71.8% in African-Americans to 86.5% in Caucasians as well as multiple race or other [11]. In-hospital exclusive rates ranged from 15.1% in Hispanics to 52.5% in Caucasians [11]. In-hospital rates reported for the participants' birthing hospital averaged 90% for initiation of breastfeeding and 41% for exclusive breastfeeding in 2001 [12]. The highest in-hospital initiation rate for the same hospital and year was 93% for Asian/Pacific Islanders. The highest recorded exclusivity rate for this hospital (in 2000) was 46% in non-Hispanic whites [12]. Specific rates for duration of breastfeeding for 3, 6 and 12 months are not available for Orange County or the birthing hospital.

## Methods

The pilot program was implemented and audited within a family practice setting consisting of nine physicians and three nurse practitioners. Three of the physicians practiced low-risk obstetrics, and one of the nurse practitioners was a board certified lactation consultant. The practice followed the AAP's 10 Steps [7]. The surrounding community was urban and multicultural with a substantial high socio-economic class population (Orange County, California).

For this pilot program, a total of 42 mothers were recruited in a 6-month time frame (beginning late September 2000), but 9 dropped out due to pregnancy-related reasons such as spontaneous miscarriage. None requested to be removed from the study, nor were any excluded from participating. The authors are not aware of any prospective candidate declining to participate, although medical assistants usually initially approached potential participants as they checked them in for their appointment. The project was reviewed and approved by the Orange County Breastfeeding Coalition and the medical directors of the practice. As the project was an audit of an educational pilot program and regarded as a quality assurance program, it was not submitted to an Institutional Review Board.

**Figure 1:**
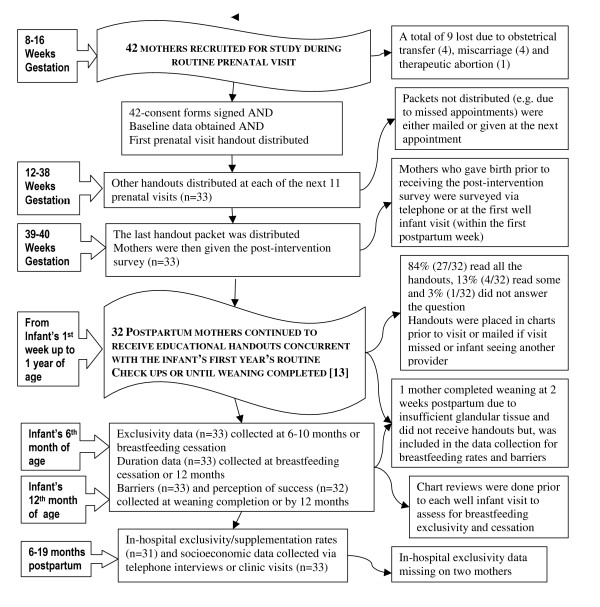
Flow chart detailing the methods and sequence of events surrounding the implementation of the program and its audit.

Mothers were usually recruited at the first prenatal visit but were accepted up to the second or third visit and prior to 16 weeks of pregnancy. Any mother attending this practice as an obstetrical patient was a candidate. Once recruited, consent forms were obtained and mothers were asked to state their breastfeeding duration and exclusivity goals. At the same time, mothers were also given the first breastfeeding educational handout and instructed to read this and subsequent handouts while waiting for their provider. The handouts were distributed at each prenatal visit and at each well baby checkup during the first year, unless breastfeeding ceased earlier. Generally well-baby visits occurred at 1 week, 2 weeks, 2 months, 4 months, 6 months and 12 months. When necessary, missed packets were either distributed at the next appointment or, occasionally, mailed (Figure 1).

The mother's physician was instructed to emphasize certain key points during each visit so that if the mother had any questions, she could ask her physician at that time. Just prior to or within the first week of birth (post-intervention) her goals were reassessed. Percent change from baseline was used to measure increases in breastfeeding goals. All unsure or indefinite answers were eliminated from analysis. Actual exclusive and overall duration breastfeeding rates were collected via questionnaires that also asked the mother if she was able to meet her goals. Exclusive breastfeeding was initially defined as "feeding your baby only your milk."

Once breastfeeding ceased or the infant turned a year of age, maternal perception of success was evaluated. Breastfeeding duration was assessed via chart review after every well-baby visit or earlier if it was learned that breastfeeding had ceased. Questionnaires were mailed or phone calls were made to collect data as needed (i.e. data was missing, mother had moved or transferred to another health care provider). Socioeconomic, work status and birthing practices, and in-hospital supplementation data were collected 6–19 months postpartum, generally via telephone interviews. The program's flow sheets, checklists and handouts can be accessed on-line at the following website: "The Breastfeeding Friendly Clinic: Breastfeeding Advice, Assistance and Advocacy" [13].

## Results

Sixty-one percent (20/33) of participants were Caucasian, 48% (16/33) had a bachelor's degree or higher, and 67% (22/33) reported an income level of US$75,000 or higher.

Originally, 9 mothers were unsure about their exclusive breastfeeding goals and one did not plan to exclusively breastfeed at all. Only 5 had goals that were less than 4 months; 16 had goals that were 4 months or more. One mother did not answer the question and one stated, "Until milk runs out."

Post-intervention, 100% (33/33) of mothers planned to exclusively breastfeed for at least some period of time. The largest increases in goals were seen in the 4–6 month group for exclusivity and in the 6–12 month group for overall duration (Table 1). There were still three mothers (9%) who were unsure of their overall duration of breastfeeding goals, but they all had definitive exclusive breastfeeding goals.

**Table 1:** Baseline goals compared to post-intervention exclusive breastfeeding and overall duration of breastfeeding goals

Exclusive Breastfeeding Goals (N = 31*)							
**Goals (Expressed in Months)**	**Unsure†**	**No**	**1–3**	**4–6**	**6**		**Total**

**Baseline**	9	1	5	5	11		22
**Post-Intervention**	0	0	5	15	11		31
**% Change**	N/A	-100	0	200	0		41

**Overall Breastfeeding Goals (N = 33)**							

**Goals (Expressed in Months)**	**Unsure†**		**1–3**	**4–6**	**6–12**	**12 or more**	**Total**

**Baseline**	10		1	6	5	11	23
**Post-Intervention**	3		0	6	13	11	30
**% Change**	N/A		-100	0	160	0	41

*Two were eliminated from analysis for missing data.

†All unsure responses were eliminated from percent analysis.

The program's actual duration of breastfeeding results were: initiation 100%, 3 months 88%, 6 months 73% and 12 months 33%. The in-hospital exclusivity rate was 61%.

When barriers to meeting goals were evaluated, mothers reported more barriers to reaching exclusivity goals than duration goals, 67% (22/33) vs. 58% (19/33). Low milk supply was reported as a barrier by 30% (10/33). Nipple soreness was reported by 79% (26/33), and rates for in-hospital supplementation for latching and hunger problems were 32% (10/31). Despite the barriers, most mothers were likely to perceive themselves as successful, and 21% (7/33) exceeded their duration goals. More specifically, 76% (25/32) perceived meeting their exclusivity goals, and 78% (25/32) perceived meeting overall duration goals. However, in actuality, 55% (18/33) of mothers met their stated exclusive breastfeeding goals, and 67% (22/33) met their declared overall duration of breastfeeding goals.

## Discussion

Some mothers remarked that they changed their goals after their babies were born because of breastfeeding difficulties like breast refusal and sucking dysfunction. Reevaluating and changing one's goals may be a positive coping mechanism employed when a mother experiences barriers that are too challenging for her to overcome.

Low milk supply was identified as the most significant barrier (and likely the most challenging) to meeting breastfeeding goals. However, all of the basics for protecting the milk supply were presented in the program, including the essentials of an adequate latch. In hindsight, latching may be an eye-hand coordination skill that may need to be discussed, demonstrated, and then practiced, particularly when mothers and babies are separated, medicated and not given an uninterrupted opportunity to self-attach at birth [2,14]. In addition, while it was recommended that mothers watch a videotape and attend a breastfeeding class at which latch was taught and demonstrated, compliance was not assessed. Nevertheless, the aforementioned rate of nipple soreness and in-hospital supplementation for latching/hunger problems supports the theory that this skill was not learned satisfactorily. Perhaps an intensive workshop to focus on latching should be offered to first-time breastfeeding mothers. Certainly, it is crucial that every postpartum nurse become trained in the basics of latching and breastfeeding management for the first few days of life [2,8]. Lastly, despite employing several lactation specialists, the birthing hospital is not Baby-Friendly.

The study had planned to measure exclusive breastfeeding rates over the first 6 months, but after the baseline evaluation, it became clear that the concept of 6 months of exclusive breastfeeding followed by complementary breastfeeding was not clear to some mothers. Thus, exclusive breastfeeding was more specifically defined on the post-intervention survey as "giving only human milk – no [infant] formula or solids like rice cereal." However, meeting this definition for a 6 month period was obtainable for only a very few mothers. This was due to in part to hospital supplementation rates and the barriers experienced by mothers (e.g. low milk supply). Furthermore, measuring exclusivity using a survey methodology that asked mothers what they practiced over the first six months of their infant's life versus using a 24-hour or monthly recall also affected rates.

This program was successful despite using handouts and counseling during routine visits; approaches found ineffective by the United States Preventive Services Task Force (USPSTF). One explanation for its success may be that, in addition to handouts, the program also incorporated the AAP's 10 Steps and a variety of other outpatient interventions. Notably, the outpatient interventions utilized have been found effective by the USPSTF and/or consistent with a 2001 literature review's findings regarding beneficial approaches [8,9]. Examples of these approaches include: face-to face guidance, employing a lactation consultant, and prenatal followed by postnatal instruction (i.e. a long-term and rigorous intervention). Moreover, one unique feature of this program was asking mothers to set goals. This may have been, in and of itself, an important component of the program, motivating mothers and leading to higher rates of breastfeeding [10].

Rates reported in this small pilot, exceeded the Healthy People 2010 overall duration of breastfeeding goals [4]. Additionally, initiation and in-hospital exclusive rates exceeded the surrounding community's [11,12]. They were not only higher than rates recorded in Orange County, California, but also specifically at the hospital where these mothers gave birth. Thirdly, the overall duration breastfeeding rates exceeded the highest rates found by the DHHS at 3, 6 and 12 months [5]. In 2003 the average U.S. rate at 3 months was 50.2% [5]. The highest 6- and 12-months rates found in any socioeconomic group or U.S. location were 58.1% in King County, Washington, at 6 months and 31.0% for Hawaii at 12 months.

The following are limitations of the program: there was a lack of a control group, maternal goals may have changed over time, independently of the program, and sometimes research participants may improve their performance simply because they are being studied.

## Conclusion

This was an audit of a small pilot educational program with a mostly privileged population; caution should be used before applying the findings. Yet comprehensive breastfeeding educational programs, such as the one outlined in this report, may have significant impacts on breastfeeding goals. Setting and meeting goals may increase duration and in-hospital exclusivity rates as well as enhance maternal self-perception and empowerment due to succeeding at their breastfeeding goals and/or experiencing a fulfilling breastfeeding relationship.

## Competing interests

The author(s) declare that they have no competing interests.

## Authors' contributions

**CMB **developed the program and wrote several of the handouts. She developed the questionnaires and collected the information. It was also her responsibility to analyze, organize and report the data. **KML **reviewed the handouts for accuracy, educated patients and recruited them. She collected questionnaires and consent forms. She also reviewed the manuscript and questionnaires for content, clarity and editorial considerations. **CS **recruited and educated patients and reviewed the manuscript for content, clarity and editorial considerations. She collected questionnaires and consent forms. All authors reviewed the final draft and approved it for publication.
